# MADS-Box Transcription Factor AGL21 Regulates Lateral Root Development and Responds to Multiple External and Physiological Signals

**DOI:** 10.1093/mp/ssu088

**Published:** 2014-08-13

**Authors:** Lin-Hui Yu, Zi-Qing Miao, Guo-Feng Qi, Jie Wu, Xiao-Teng Cai, Jie-Li Mao, Cheng-Bin Xiang

**Affiliations:** School of Life Sciences, University of Science and Technology of China, Hefei, Anhui Province 230027, China

**Keywords:** MADS, root system architecture, lateral root, *AGL21*, auxin, nitrate, sulfate.

## Abstract

Plant root system morphology is dramatically influenced by various environmental cues. The adaptation of root system architecture to environmental constraints, which mostly depends on the formation and growth of lateral roots, is an important agronomic trait. Lateral root development is regulated by the external signals coordinating closely with intrinsic signaling pathways. MADS-box transcription factors are known key regulators of the transition to flowering and flower development. However, their functions in root development are still poorly understood. Here we report that *AGL21*, an *AGL17*-clade MADS-box gene, plays a crucial role in lateral root development. *AGL21* was highly expressed in root, particularly in the root central cylinder and lateral root primordia. *AGL21* overexpression plants produced more and longer lateral roots while *agl21* mutants showed impaired lateral root development, especially under nitrogen-deficient conditions. *AGL21* was induced by many plant hormones and environmental stresses, suggesting a function of this gene in root system plasticity in response to various signals. Furthermore, AGL21 was found positively regulating auxin accumulation in lateral root primordia and lateral roots by enhancing local auxin biosynthesis, thus stimulating lateral root initiation and growth. We propose that AGL21 may be involved in various environmental and physiological signals-mediated lateral root development and growth.

## SUMMARY

MADS-box transcription factor AGL21 is responsive to several phytohormones as well as environmental cues and positively regulates auxin accumulation in lateral root primordia and lateral roots by enhancing local auxin biosynthesis, thus stimulating lateral root initiation and growth. Therefore, *AGL21* may be involved in various environmental and physiological signals-mediated lateral root development.

## INTRODUCTION

Root systems are crucial for plant survival, responsible for acquisition of water and mineral nutrients and anchorage, and contributing to competitive fitness in the changing environment (Lloret and Casero, 2002). In order to adapt to the changing environment, plant root system architecture (RSA) is highly plastic, responding to various environmental cues, such as soil matrix heterogeneity (Hodge, 2006), distribution of nutrients in the soil (Leyser and Fitter, 1998), and biotic interactions (Osmont et al., 2007). These extrinsic signals trigger intrinsic molecular mechanisms that have profound impacts on RSA through regulating cell division and cell differentiation processes within the root (Malamy and Ryan, 2001; Wolters and Jurgens, 2009). This allows the immobile plants to initiate root growth, such as root-hair formation, primary root (PR) growth, and lateral root (LR) formation, to greatly increase the total surface area and mechanical strength of the root system and allow the plant to efficiently adapt to environmental constraints (Deak and Malamy, 2005; Malamy, 2005; Ariel et al., 2010). Well-developed root systems have been reported beneficial for enhancing plant water and nutrient uptake and dehydration avoidance, thus increasing yield under soil-related stresses (Lynch, 2007; Serraj et al., 2009). The design of sustainable cropping systems with high yield can be achieved if sufficient knowledge about root development is available.

In response to diverse environmental signals, plants adjust their growth and development through the perception and integration of external signals into the signaling pathways of plant hormones, such as auxin, cytokinin, abscisic acid (ABA), and jasmonic acid (JA) (Lopez-Bucio et al., 2003; Malamy, 2005; De Smet et al., 2006b; Kazan, 2013). As physiological signals and hormonal factors interact with each other to modulate root development in which auxin, its polar transport, and local biosynthesis appear to have emerged as central regulators (De Smet et al., 2006a; Fukaki and Tasaka, 2009; Lavenus et al., 2013). Recent studies have shown that proper auxin transport, biosynthesis, and signaling control various steps of LR development from priming to initiation, patterning, and emergence (Fukaki et al., 2007; Lavenus et al., 2013).

Transcription factors (TFs) are known to be important for root development (Montiel et al., 2004). The MADS (*MCM1*/*AGAMOUS*/*DEFICIENS*/*SRF*) box TF family genes play important roles in controlling plant and animal development (Messenguy and Dubois, 2003). These TFs have been classified into two types (type I and type II) based on sequence relationships and structural features (Alvarez-Buylla et al., 2000b). There are 45 type II genes, which are also referred to as MIKC TFs for the four domains (M, I, K, C) they contain (Alvarez-Buylla et al., 2000b; Kaufmann et al., 2005). Plant MIKC TFs have been mostly characterized as regulators of the flowering time (Samach et al., 2000) and flower, seed, and fruit development (Saedler et al., 2001; Pinyopich et al., 2003; de Folter et al., 2006; Ripoll et al., 2011). However, some of these TFs are also expressed in various organs and vegetative tissues, such as endosperm, pollen, guard cells, trichomes, and roots, where they may have more specific functions (Alvarez-Buylla et al., 2000a; Arora et al., 2007). It has been reported that at least 50 MADS-box genes are expressed in *Arabidopsis* roots (Burgeff et al., 2002; Parenicova et al., 2003). However, the functions of these TFs in roots are largely unknown. Recently, *XAL1/AGL12* and *XAL2/AGL14* were reported to have roles in regulating PR growth (Tapia-Lopez et al., 2008; Garay-Arroyo et al., 2013). *ANR1* is so far the only member of the family reported as being involved in LR development (Zhang and Forde, 1998). Gan et al. (2005) compared the responsiveness of *ANR1* and 11 other root-expressed MADS-box genes to the availability of nitrogen (N), phosphorus (P), and sulfur (S), and found that seven of the them responded to N in a manner similar to *ANR1* but less strongly, suggesting possible roles of these genes in nutritional regulation of LR growth.

In this study, we report that the MADS-box gene *AGL21* is involved in LR development. *agl21* mutant alleles had fewer and shorter LRs, while overexpression of *AGL21* increased both the number and length of LRs compared with the wild-type. Further analyses showed that *AGL21* was expressed in silique, flower, and seed, but mainly in roots, with higher levels during LR formation, from young lateral root primordia (LRPs) to emerged LRs. Furthermore, *AGL21* responds to many hormones, including indole-3-acetic acid (IAA), methyl jasmonate (MeJA), ABA, as well as many environmental stresses such as nutrient starvations. More importantly, our data show that AGL21 positively regulates auxin accumulation and cell division activities in LRPs and LRs, suggesting that AGL21 is likely to regulate LR formation and growth by integrating multiple external and physiological signals into auxin signaling.

## RESULTS

### AGL21 Is a Positive Regulator of LR Development

**Figure 1:**
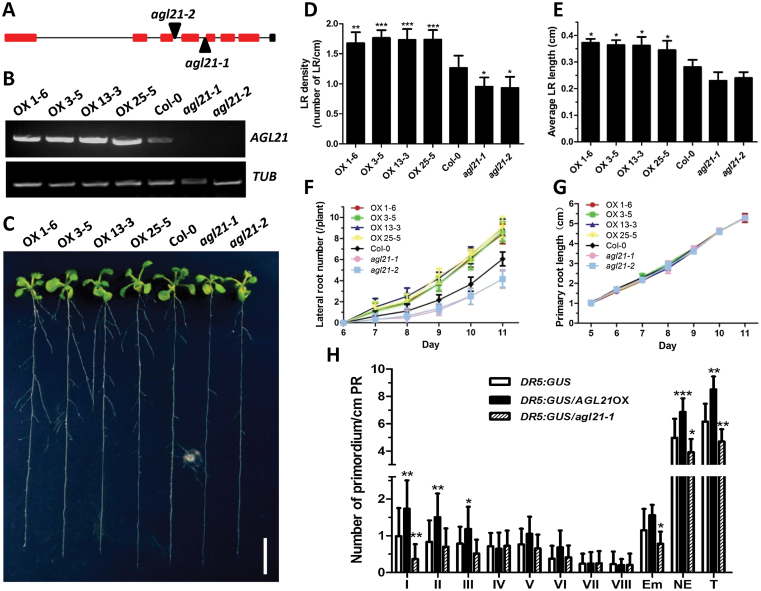
AGL21 Is Involved in LR Development. The seeds were germinated for 5 d on MS medium, and the seedlings were then transferred to MS medium for vertical growth. **(A)** *AGL21* gene structure with the sites of T-DNA insertion. Squares correspond to exons while lines represent introns. **(B)** *AGL21* transcript levels in the transgenic lines and mutants by RT–PCR analysis. *TUBULIN* (*TUB*) was used as the internal control. **(C)** Root systems of 12-day-old *35S::AGL21*, *agl21* mutants and wild-type (WT) (Col-0) seedlings (bar = 1cm). **(D)** Density of visible LRs of 12-day-old plants. Density of visible LRs is defined as visible LR number per cm PR. Values are mean ± standard deviation (SD) of three independent experiments each containing 15–20 plants per genotype. Asterisks denote Student’s *t*-test significance compared with WT plants: * *P* < 0.05; ** *P* < 0.01; *** *P* < 0.001. **(E)** Average LR length of 12-day-old plants. Average LR length is defined as the ratio of total LR length over LR number. Values are the mean ± SD of three independent experiments each containing 15–20 plants per genotype. Asterisks denote Student’s *t*-test significance compared with WT plants: * *P* < 0.05. **(F, G)** LR and PR growth curves of WT, *agl21* mutants, and *35S::AGL21* plants. **(H)** Numbers of LRP of 8-day-old seedlings at given stages. Stages of primordia were based on the classification by Malamy and Benfey (1997). Values are mean ± SD of three independent experiments each containing 15 plants per genotype and asterisks denote Student’s *t*-test significance compared with WT plants: * *P* < 0.05; ** *P* < 0.01; *** *P* < 0.001. NE, non-emerged LR; E, emerged LR; T, NE + E

To gain insight into the function of *AGL21*, we generated *35S::AGL21* transgenic *Arabidopsis* plants, and obtained two T-DNA insertion mutants: CS118325 (*agl21-1*) and GK_157C08 (*agl21-2*) (Figure 1A and 1B). The effect of *AGL21* on the development of the root system was examined using *35S::AGL21* (OX), *agl21* mutants, and the wild-type plants vertically grown on Murashige and Skoog (MS) medium. After 12 d of growth, the *AGL21* overexpression plants produced significantly better-developed root system than wild-type plants with higher LR density and longer average LR length, while the mutants produced much less and shorter LRs than wild-type plants (Figure 1C–1E). Time-course data also showed that *AGL21* overexpression plants possessed an apparent advantage over LR development compared with the wild-type and mutant plants (Figure 1F). However, no obvious differences in PR length were observed between the *AGL21* overexpression plants, wild-type, and the mutants (Figure 1G).

For more detailed analysis, we introduced *DR5:GUS* reporter (Ulmasov et al., 1997) into *35S::AGL21* and *agl21* mutant background by crossing and performed quantitative LPR and LR growth analysis by quantifying the number of GUS-stained loci of primordia and emerged LRs of 8-day-old seedlings. The results showed that *AGL21*-overexpressing plants had significantly increased GUS-stained LRP loci compared with the wild-type and mutant plants (Supplemental Figure 1). Further analysis demonstrated that *AGL21* mainly affected the early stages of LRP development. The number of LRP at I to III stages was much higher in the *AGL21*-overexpressing plants, while the corresponding figures in the *agl21* mutant background were much lower. Consequently, overexpression of *AGL21* increased the numbers of non-emerged LR as well as emerged LR (Figure 1H). On the contrary, *AGL21* knockout negatively affected LR development (Figure 1C–1H). These results suggest that AGL21 is a positive regulator of LR initiation and growth.

### Expression Pattern of *AGL21* and Subcellular Localization of AGL21 Protein

**Figure 2:**
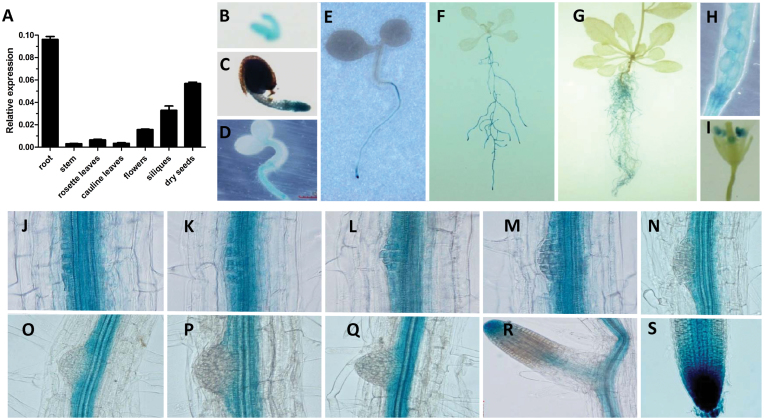
*AGL21* Expression Pattern. **(A)** Analysis of the *AGL21* expression pattern in different organs by qRT–PCR. *UBQ5* was used as an internal control. Values are mean ± SD of three replica experiments. **(B–I)** The expression pattern of *AGL21*, as revealed by promoter–GUS fusion analyses in *pAGL21::GUS* transgenic seedlings. GUS activity was observed in embryo (B), seedling of 2-day-old (C), seedling of 3-day-old (D), seedling of 4-day-old (E), seedling of 14-day-old (F), seedling of 35-day-old (G), silique (H), and flower (I). **(J–S)** The *pAGL21::GUS* expressed in LRPs at stages I to VIII (J–Q), emerged LR (R), and PR tip (S). Eight-day-old *pAGL21::GUS* transgenic lines were used for GUS reaction for 12h.

In order to investigate the expression patterns of the *AGL21* in more detail in *Arabidopsis*, we first measured its expression levels by quantitative real-time PCR (qRT–PCR) in various organs. We detected the expression of this gene mostly in root, flowers, siliques, and dry seeds, with the strongest expression in root (Figure 2A). This result was further confirmed by using plants expressing a GUS reporter gene placed under the control of the 2.6-kb *AGL21* promoter region (*pAGL21::GUS*). Histochemical analyses showed similar results to qRT–PCR. *AGL21* was primarily expressed in the root of seedlings from germination to mature stage (Figure 2C–2G), and the expression of *AGL21* in the root is mostly confined to central cylinder of the whole PR with much higher expression levels in the root tip and meristem (Figure 2E). In addition, *AGL21* transcript was also detected in embryo and silique, and its expression in flower was confined to stamen-anther (Figure 2B, 2H, and 2I). More careful observation revealed that *AGL21* was expressed at a higher level during LR formation, from young LRPs with one single cell layer to about three to four cell layers (Figure 2J–2M). However, hardly any expression was detected in LRPs at stages V to VIII (Figure 2N–2Q), which is consistent with the result that the effects of *AGL21* on LR initiation was mainly on the early stages of LRP (Figure 1F). In emerged LR, its expression was focused on the apex and proliferative zone (Figure 2R). In the PR tip, *AGL21* was expressed in all cell layers and had the strongest expression in the quiescent center (QC) area (Supplemental Figure 2). These results agree with previous reports (Burgeff et al., 2002; Parenicova et al., 2003). Taken together, our results imply that *AGL21* may be involved in LR development and consistent with the phenotypes we detected in the root of *AGL21* overexpression and knockout plants.

**Figure 3:**
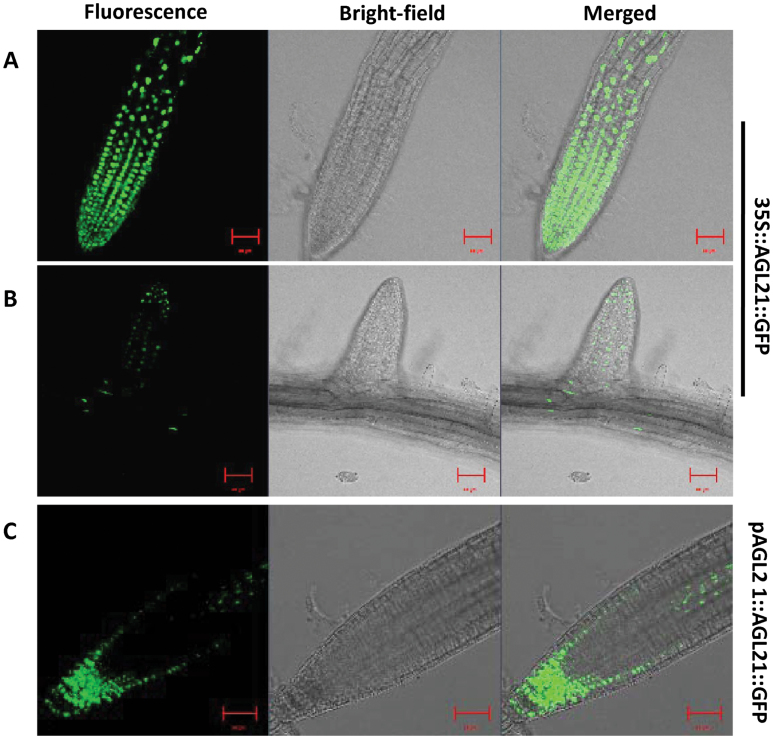
Subcellular Localization of AGL21 Protein. **(A, B)** Fluorescence in the root cells of transgenic plants expressing AGL21–GFP under the control of the CaMV 35S promoter (bar = 50 μm). **(C)** Fluorescence in the root cells of transgenic plants expressing AGL21–GFP under the control of the 3.6-kb *AGL21* promoter (bar = 50 μm).

To investigate the localization of AGL21 protein in plant cell, we generate transgenic plants expressing green fluorescent protein (GFP) fusion protein under the control of 35S promoter (*35S::AGL21::GFP*) and its own promoter (*pAGL21::AGL21::GFP*). Figure 3A and 3B show that AGL21 protein targeted to the nucleus of the root cells of both PR and LR. Under the control of its own promoter, AGL21 protein is strongly expressed in the PR tip region, especially in the QC area. In the meristematic region, AGL21 protein is mainly restricted to the epidermal cell layers, while, in the elongation zone, AGL21 protein is focused in the central cylinder (Figure 3C). These data agree well with the gene expression pattern of *AGL21* and the function of AGL21 in root development.

### *AGL21* Is Responsive to Multiple Plant Hormones and Nutrient Deficiency

**Figure 4:**
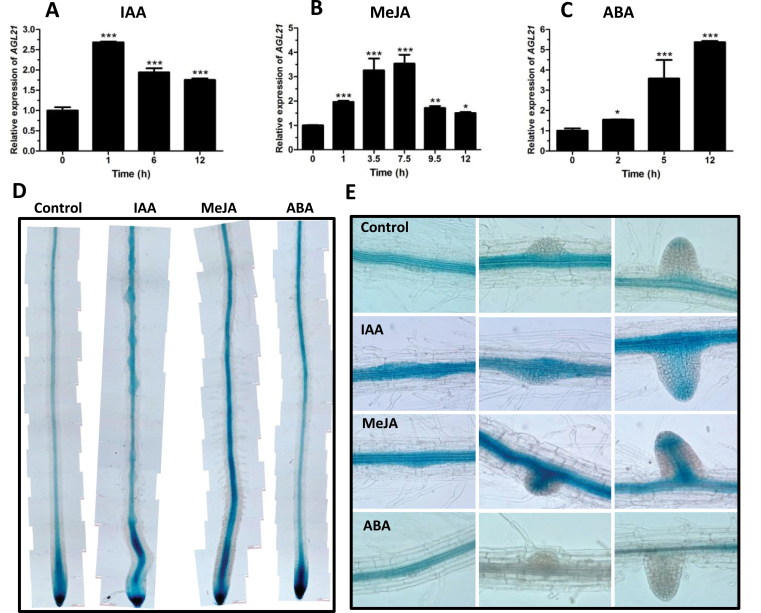
*AGL21* Expression Is Regulated by Hormones. **(A–C)** qRT–PCR analyses of *AGL21* expression in wild-type seedlings during the time course after IAA (A), MeJA (B), or ABA (C) treatment. Eight-day-old Col-0 seedlings were incubated in MS liquid cultures with 10 μM IAA, 50 μM MeJA, and 20 μM ABA, respectively, and whole seedlings were harvested at indicated time points for RNA extraction and qRT–PCR analyses. The transcript levels of *AGL21* were normalized to the *UBQ5* expression. Values are mean ± SD and asterisks denote Student’s *t*-test significance compared with untreated plants: * *P* < 0.05; ** *P* < 0.01; *** *P* < 0.001. **(D, E)** IAA, MeJA, and ABA-induced *pAGL21::GUS* expression in the primary root (D), LRP, and LR (E). Seven-day-old seedlings of *pAGL21::GUS* transgenic line grown on MS agar medium were transferred either to hormone-free MS agar medium or to MS agar medium supplemented with 10 μM IAA, 10 μM MeJA, or 10 μM ABA for 1 d, respectively. The seedlings were harvested for GUS staining for 8h.

Several types of *cis*-acting elements, including auxin response element (AuxRE), ABA response element like (ABRE-like), G-box, GCC-box like, and JA-responsive *cis*-element (JARE) were found in the *AGL21* promoter (Supplemental Figure 2), implicating that *AGL21* could respond to various plant hormones and environmental stresses. We thus performed qRT–PCR to test this. The results showed that the expression of *AGL21* was indeed up-regulated by IAA, MeJA, and ABA (Figure 4A–4C). To confirm these results, we treated the *pAGL21::GUS* reporter line with IAA, MeJA, and ABA. GUS staining showed that IAA and MeJA clearly induced the expression of *AGL21* in the meristematic and elongation zones of PRs (Figure 4D). IAA and MeJA could enhance the expression of *AGL21* in early stages of LPRs and emerged LRs and even the later stages of LRPs, where it was not expressed without hormone treatments (Figure 4E). The response of *AGL21* expression to IAA was particularly intense in LRPs (Supplemental Figure 3). Interestingly, ABA could induce *AGL21* expression in the PR tips, meristematic zone, and elongation zone (Figure 4D). However, in the middle and upper differentiation zone, GUS activity was diminished (Figure 4E). It should be noted that, in roots, *AGL21* was preferentially enhanced by IAA and MeJA in the central cylinder, root tips, and LRPs.

**Figure 5:**
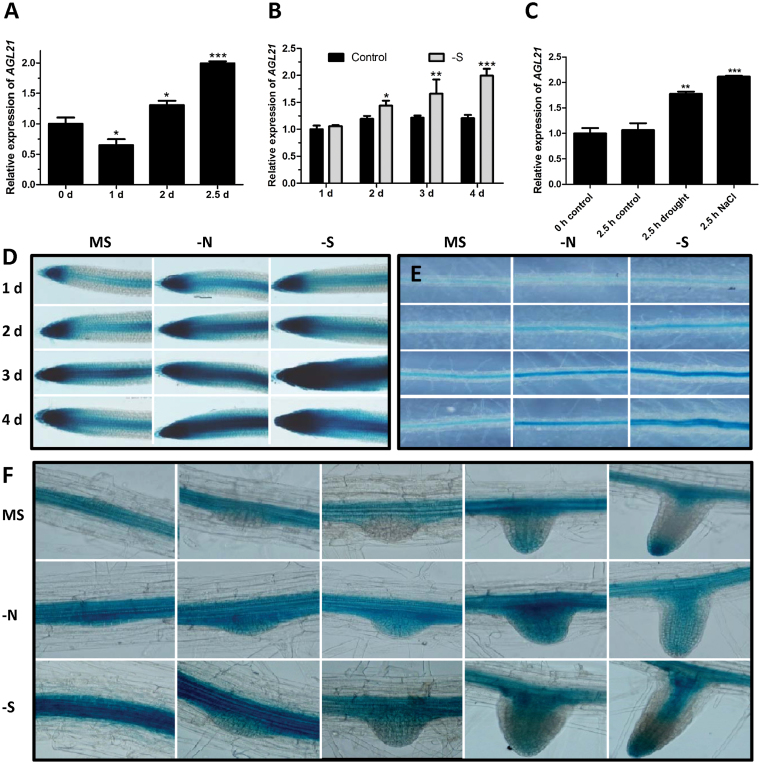
Response of *AGL21* to Multiple Environmental Stresses. **(A)** Response of *AGL21* to N starvation. Seven-day-old Col-0 seedlings were transferred to N-free nutrient solution and harvested at indicated time points for RNA extraction and qRT–PCR analyses. **(B)** Response of *AGL21* to S starvation. Seven-day-old Col-0 seedlings were transferred to S-free agar medium for vertical growth. Complete nutrient medium was used as control. Roots were harvested at indicated time points for RNA extraction and qRT–PCR analyses. **(C)** qRT–PCR analyses of *AGL21* expression in 8-day-old Col-0 seedlings after 2.5h of drought or 120mM NaCl treatment. The transcript levels of *AGL21* were normalized to the *UBQ5* expression. Values are mean ± SD of three replica experiments and asterisks denote Student’s *t*-test significance compared with the 0-h control plants: * *P* < 0.05; ** *P* < 0.01; *** *P* < 0.001. **(D–F)** Effects of N deprivation and S deprivation on *pAGL21::GUS* expression in the roots. Expression of *AGL21* is induced by N deprivation and S deprivation in the PR tips (D), differential zone (E) after 1–4 d of N or S starvation, and LRPs after 3 d of N or S starvation (F). Five-day-old *pAGL21::GUS* transgenic seedlings grown on MS medium were transferred to N- or S-free medium for 1–4 d and seedlings were harvested at the indicated time points for GUS staining for 8h.

To analyze the response of *AGL21* to external stresses, we performed qRT–PCR and GUS staining of *pAGL21::GUS* reporter line to examine the response of *AGL21* to different stresses. Figure 5A and 5B indicate that *AGL21* expression was induced by N or S deprivation. Interestingly, *AGL21* expression was also responsive to drought and NaCl treatment (Figure 5C). Moreover, results of GUS staining demonstrated that the expression of *AGL21* was clearly up-regulated in the root after 1–4 d of N or S starvation as shown in Figure 5D and 5E. Surprisingly, we found *AGL21* was strongly induced in all stages of LRP and LRs after 3 d of N starvation, even the later stages of LRP where *AGL21* did not express under normal conditions (Figure 5F). Induced expression of *AGL21* in LRPs was also detected after 3 d of S deprivation, although less strongly compared with N deprivation treatment (Figure 5F). These results indicate that AGL21 is an important TF, at which multiple hormones and stress signals are converged to regulate LR development.

### AGL21 Is an Important Factor to Sustain LR Development under Low-N Conditions

**Figure 6:**
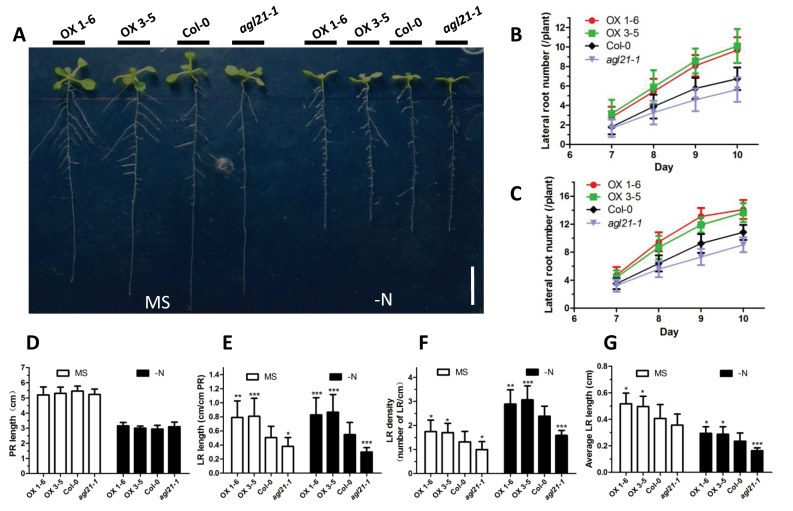
AGL21 Is Important for Sustaining LR Development under Low-N Conditions. Five-day-old seedlings grown on MS medium were transferred to N-free medium and grown vertically. During the vertical growth stage, the root morphological parameters were measured. **(A)** Phenotype of 11-day-old seedlings of *AGL21*-overexpressing, Col-0, and *AGL21* knockout (bar = 1cm). **(B, C)** Numbers of visible lateral roots of *AGL21*-overexpressing, Col-0, and *AGL21* knockout plants on MS medium (B) and N-free medium (C) from the 7th day to the 10th day. Values are mean ± SD of three independent experiments each containing 15–20 plants per genotype. **(D)** Average PR length of the 11-day-old plants. Values are mean ± SD of three independent experiments each containing 15–20 plants per genotype. **(E–G)** Length of visible LRs per cm PR length (E), LR density (F), and average LR length (G). Average LR length defined as the ratio of total LR length over LR number. Values are mean ± SD of three independent experiments each containing 15–20 plants per genotype and asterisks denote Student’s *t*-test significance compared with the wild-type plants: * *P* < 0.05; ** *P* < 0.01; *** *P* < 0.001.

To further study whether AGL21 is involved in environmental signals regulating LR development, we analyze the role of AGL21 in LR development in response to N availability. Six-day-old seedlings of the mutant and *AGL21*-overexpressing plants were transferred from MS to N-free medium and vertically grew. Visible LR number was monitored in the following days. The results clearly demonstrated that there was a significant increase in LR number per plant in the *AGL21*-overexpressing plants compared with the wild-type plants under both N-free and -rich conditions. In contrast, the LR number of the *agl21* mutant was significantly reduced (Figure 6A–6C). However, no obvious difference was observed in the PR length (Figure 6D). Meanwhile, we also found that AGL21 affected the LR elongation under both N-rich and -free conditions. There was a slight increase in LR length per centimeter (cm) PR under the N-free conditions compared with the N-rich conditions, except in the mutant, in which the corresponding figure decreased instead (Figure 6E). However, LR length per centimeter PR is related with both LR density and LR length. Thus we checked the LR density and average LR length. Compared with the wild-type, LR density and average LR length of *AGL21* overexpression plants increased by 29.6%–33.0% and 22.1%–27.2%, respectively, under N-rich conditions, 21.1%–28.4% and 22.2%–25.4%, respectively, under N-free conditions. In *agl21* mutant plants, LR density decreased by 21.1%, and average LR length decreased by 12.5.0% under N-rich conditions, but, under N-free conditions, the corresponding figures of LR density and average LR length reduced by 25.1% and 30.4% compared with wild-type, respectively (Figure 6F and 6G). These results imply that AGL21 is important to both LR initiation and LR elongation, but mainly affecting LR elongation under N-restricted conditions.

### Auxin Can Rescue the Phenotype of *agl21* Mutant

**Figure 7:**
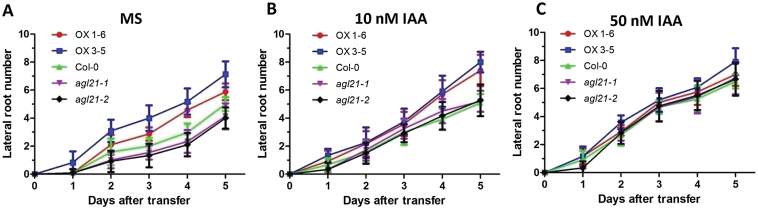
LR Phenotype Is Rescued by Exogenous IAA. Five-day-old plants grown on MS vertical agar plates were transferred to MS agar plates containing different concentrations of IAA to grow vertically for 6 d. Values are mean ± SD of three independent experiments each containing 15 plants. **(A)** Time course of LR development on MS medium. **(B)** Time course of LR development on MS medium supplemented with 10nM IAA. **(C)** Time course of LR development on MS medium supplemented with 50nM IAA.

To test whether AGL21 regulates LR development through changing auxin concentration in the LRPs and LRs, we assayed the root phenotype by adding exogenous IAA in the medium. Without exogenous IAA, *AGL21*-overexpressing plants developed more LRs while the *agl21* mutant plants had much fewer LRs compared with the wild-type plants (Figure 7A). However, after adding 10nM IAA, the differences in LR number between the wild-type and knockout plants diminished (Figure 7B). When exogenous IAA concentration was increased to 50nM, no differences in root number were observed (Figure 7C). These results suggest that AGL21 may regulate LR development by altering endogenous auxin concentration.

### AGL21 Regulates Auxin Accumulation in the LRPs by Affecting Local Auxin Biosynthesis

**Figure 8:**
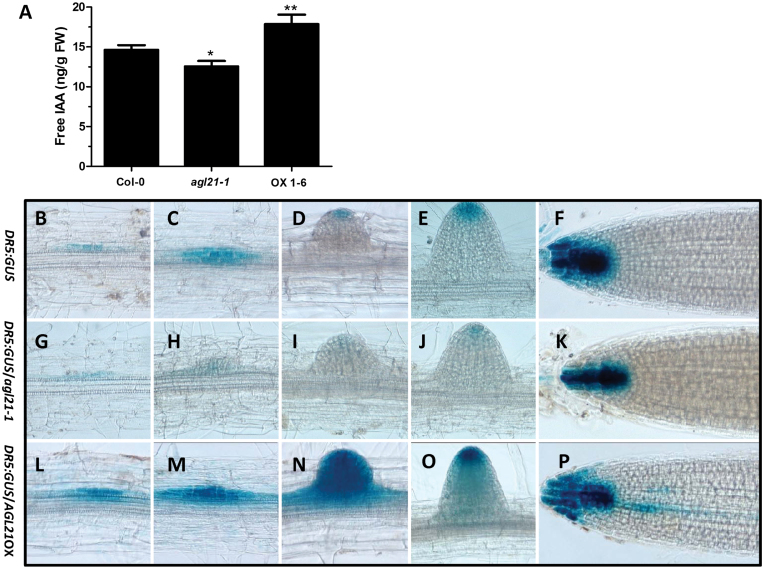
AGL21 Regulates Auxin Accumulation in the LRPs and LRs. **(A)** Quantification of free IAA content in the root of 9-day-old plants. Values are mean ± SD of three replica experiments and asterisks denote Student’s *t*-test significance compared with the wild-type plants: * *P* < 0.05; ** *P* < 0.01. **(B–F)** Expression of *DR5:GUS* (*N* = 20 plants) in three different stages of LRP (B–D), LR, and PR tips (E, F) of Col-0 plants. Nine-day-old plants grown on MS medium were used for GUS staining for 6h. **(G–K)** Expression of *DR5:GUS* in three different stages of LRP (G–I), LR, and PR tips (J, K) of *agl21* knockout plants. **(L–P)** Expression of *DR5:GUS* in three different stages of LRP (L–N), LR, and PR tips (O, P) of *AGL21*-overexpressing plants.

The LR phenotypes of *agl21* mutant and the overexpression lines implicate that root auxin level might be affected. To verify whether endogenous auxin content was changed, we measured endogenous IAA in the root. The results in Figure 8A show that IAA content was significantly increased in the overexpression line and reduced in the knockout mutant compared with that in the wild-type.

In addition, we introduced the auxin-responsive *DR5:GUS* marker line into *AGL21*-overexpressing and *agl21* knockout background by crossing to indicate endogenous auxin distribution in roots (Ulmasov et al., 1997). In the wild-type background, *DR5:GUS* reporter was stained in foci in LRPs and tips of PR (Figure 8B–8F). In pre-emerging and young wild-type LRs, *DR5:GUS* was expressed exclusively in the apex (Figure 8D and 8E). Notably, *DR5:GUS* in the LRPs and young LR was markedly repressed in *agl21* mutant background (Figure 8G–8J). On the other hand, a dramatically increased level of GUS staining was observed in the LRPs and emerged LRs in *AGL21*-overexpression background (Figure 8L–8O), especially in the emerging LRs (Figure 8N). Overexpression of *AGL21* did not apparently alter *DR5:GUS* expression in the PR tips (Figure 8P). However, the expression was slightly reduced in the PR tips of *agl21* mutant (Figure 8K). Moreover, GUS activities were clearly strengthened in the leaves. In *agl21* mutant background, the *DR5:GUS* expression was limited to the margin of young leaves but, in the *AGL21* overexpression background, GUS activity extended to veins (Supplemental Figure 4). From these results, it appears that AGL21 acts as a positive regulator of auxin accumulation in the LRPs and LRs, thus resulting in more LRP initiation and faster LR growth.

**Figure 9:**
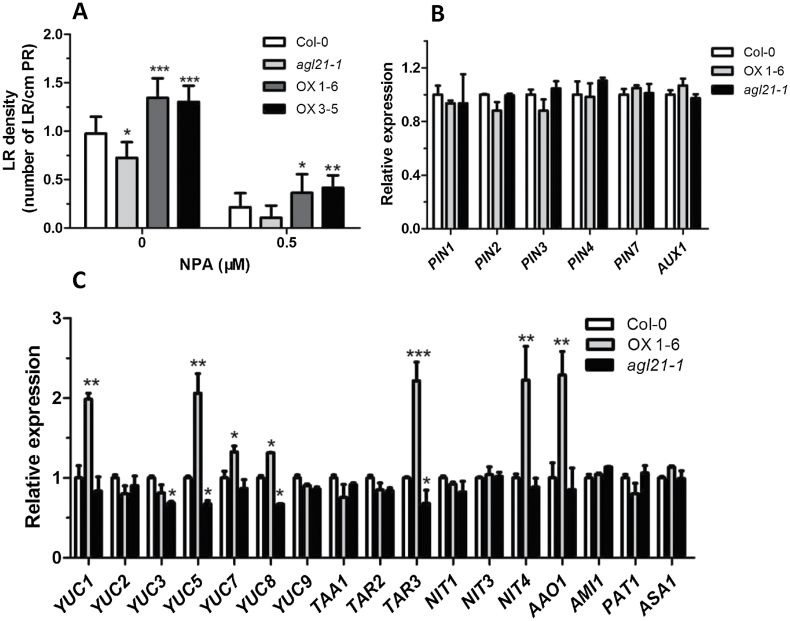
AGL21 Increases Auxin Accumulation in the Root through Local Biosynthesis. **(A)** Effects of auxin transport inhibitors NPA on LR initiation in wild-type, *AGL21*-overexpressing and mutant plants. Five-day-old seedlings were transferred to medium supplemented with DMSO alone or the auxin transport inhibitor NPA (0.5 μM) dissolved in DMSO. After 7 d of growth, the LRs produced in the new growth were counted on 30 seedlings. Values are mean ± SD of three replica experiments and asterisks denote Student’s *t*-test significance compared with the wild-type plants: * *P* < 0.05; ** *P* < 0.01. **(B, C)** Relative expression levels of auxin transport genes (B) and auxin biosynthesis genes (C) in the roots of 9-day-old plants. The transcript levels of auxin transport or biosynthesis genes were normalized to the *UBQ5* expression. The expression levels of each gene in the wild-type were set as 1.0. Values are mean ± SD of three replica experiments and asterisks denote Student’s *t*-test significance compared with the wild-type plants: * *P* < 0.05; ** *P* < 0.01.

Tempo-spatial auxin accumulation is regulated by local auxin biosynthesis or/and polar transport. In order to elucidate which pathway AGL21 is involved in regulating auxin accumulation in the root, we firstly treated the seedlings with auxin transport inhibitor N-1-naphthylphthalamic acid (NPA). Results showed that NPA severely reduced LR initiation in wild-type plants and *agl21* mutant plants compared with plants grown on NPA-free medium, as previously demonstrated (Reed et al., 1998). However, the *AGL21* overexpression plants developed more LRs even after transfer to medium containing NPA (Figure 9A). These results indicate that the function of AGL21 in LR development is independent of polar auxin transport. The expression levels of auxin transport genes in the root also support this conclusion (Figure 9B). We then analyzed the expression levels of many auxin biosynthesis genes, including YUCCA family, TAA1 family, NIT family, and several other genes. Three of these genes, including *YUC5*, *YUC8*, and *TAR3*, were found to be significantly up-regulated in *AGL21*-overexpressing plants and down-regulated in the mutant. However, expression levels of *YUC1*, *YUC7*, *NIT4*, and *AAO1* increased significantly in the root of *AGL21* plants, but were not so significantly down-regulated in the mutant (Figure 9C). These data indicate that AGL21 may increase auxin content in LRs and LRPs via local auxin biosynthesis.

### AGL21 Promotes Cell Division Activities in LRs and LRPs

**Figure 10:**
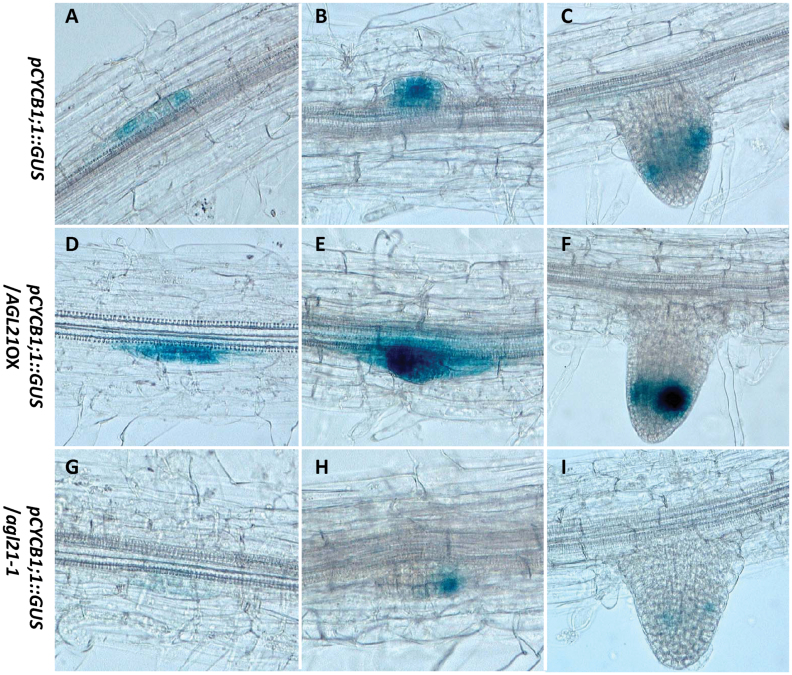
AGL21 Affects *pCYCB1;1::GUS* Expression in the LRPs and LRs. Eight-day-old seedlings were harvested for GUS staining. The seedlings were immersed in GUS staining buffer and applied vacuum for 2min, and then incubated at 37°C overnight. **(A–C)** *pCYCB1;1::GUS* expression in LRPs (A, B) and LR (C) of Col-0 background seedlings. **(D–F)** *pCYCB1;1::GUS* expression in LRPs (D, E) and LR (F) of *AGL21*-overexpressing background seedlings. **(G–I)** *pCYCB1;1::GUS* expression in LRPs (G, H) and LR (I) of *agl21* knockout background seedlings.

In order to determine whether AGL21 regulates cell division during the LR development, we introduced the *pCYCB1;1::GUS* reporter into the *agl21* mutant and *35S::AGL21* background through genetic crossing. The *pCYCB1;1::GUS* marks the cell divisions in pericycle during LR initiation and serves as a good marker to visualize the site of LRP initiation and development (Beeckman et al., 2001; Himanen et al., 2002). Results in Figure 10 indicate that AGL21 positively regulates *CYCB1;1* expression in the root. Specifically, in the *AGL21*-overexpressing background, GUS was strongly expressed in the LPRs (Figure 10D and 10E) and LR tips (Figure 10F) compared with that in the wild-type background (Figure 10A–10C). However, GUS expression was only found weakly in LPRs and LR tips of the *agl21* mutant (Figure 10G–10I), implicating impaired initial anticlinal division of pericycle cells leading to LRP initiation and LR growth in the mutant. Therefore, we propose that AGL21 positively regulates cell division activities in the LRPs and LRs, thus promoting LR development to some extent.

## DISCUSSION

### Expression Pattern of *AGL21* Supports Its Role in LR Development

The MADS-box gene family is generally subdivided into several well-defined monophyletic clades with typical similar expression pattern and highly related function (Becker and Theissen, 2003). In *Arabidopsis*, *AGL17*, *AGL21*, *ANR1*, together with *AGL16* belong to the *AGL17* clade, which are preferentially expressed in roots (Alvarez-Buylla et al., 2000a; Burgeff et al., 2002). *ANR1* is the only gene in this clade known for being involved in nitrate stimulated LR development (Zhang and Forde, 1998). In *Oryza sativa*, four of the five *AGL17*-like clade genes are expressed in the central cylinder of roots, indicating potential functions in root development (Puig et al., 2013).

Like *ANR1*, *AGL21* is primarily expressed during LR formation and embryogenesis. *In situ* hybridization experiments showed that *AGL21* expressed in the central cylinder in the differentiated zone of the PR and young LPRs up to stage III or IV as well as emerged LRs. *AGL21* was also detected in embryos from the globular stage up to the torpedo stage (Burgeff et al., 2002). In this study, we used qRT–PCR and *pAGL21::GUS* reporter line to analyze the expression pattern of *AGL21*. Our results not only agree with previous reports, but also revealed the spatiotemporal expression pattern (Figure 2), which supports that AGL21 plays an important role in LR initiation and growth.

### *AGL21* Regulates LR Initiation and Growth through Increasing Auxin Accumulation and Promoting Cell Division in the LRPs and LRs

The expression pattern of *AGL21* implicated that it may be involved in LR development. Indeed, our subsequent study demonstrated that AGL21 is important for LR initiation and growth. Overexpression of *AGL21* increases LR number and length. In contrast, *AGL21* knockout results in less and shorter LRs (Figures 1 and 6). Further analysis of the GUS staining of *DR5:GUS* reporter plants in *AGL21*-overexpressing and knockout genetic background shows that AGL21 positively regulates LRP initiation, especially the I and II stage LRPs (Figure 1H). These results are in agreement with the expression pattern of *AGL21* in central cylinder and young LRPs.

During LR development, auxin accumulation is one of the most important events for LR initiation as well as post-initiation events including emergence (Benkova et al., 2003; Peret et al., 2009). Our results suggest that AGL21 promotes LR development through increasing auxin accumulation during LRP initiation and in newly emerged LRs. At first, expression level of *AGL21* dramatically affected LRP initiation and LR growth (Figures 1 and 6)—a process depending on the auxin-mediated establishment and activity of a new meristem (Himanen et al., 2002; Osmont et al., 2007; Nibau et al., 2008). Furthermore, the changes in auxin concentration in both LRPs and young LRs of *AGL21* overexpression and knockout plants were clearly confirmed by both localization of *DR5:GUS* activity and IAA content measurement (Figure 8). Finally, exogenous IAA was able to rescue the phenotypes of *agl21* mutant plants (Figure 7). Therefore, we propose that AGL21 affects auxin homeostasis in the LRPs and LRs in two different ways, either by increasing polar auxin transport to the initiated primordia or more likely by enhancing the local auxin biosynthesis of newly formed LRPs and LRs according to the ‘fountain’ model proposed by Benkova et al. (2003). The auxin transport inhibitor NPA can arrest LR development through blocking auxin redistribution in the root (Casimiro et al., 2001). However, *AGL21*-overexpressing plants still developed more LRs after NPA treatment and the transcript level of *AGL21* did not affect the expression levels of auxin transport genes in the root (Figure 9A and 9B), indicating that AGL21’s promoting LR development may be not through affecting auxin transport. Instead, several auxin biosynthesis pathway genes were found up-regulated in the roots of *AGL21*-overexpressing plants and down-regulated to some extent in the mutant roots (Figure 9C). Therefore, taken together, our data indicate that AGL21 can enhance local auxin biosynthesis in the root to regulate LR initiation and growth.

Cell cycle activation and activity during early LR initiation are known to be regulated by auxin (Stals and Inze, 2001; Himanen et al., 2002). Thus, we crossed *pCYCB1;1::GUS* line with *AGL21*-overexpressing and knockout plants and analyzed the cell cycle activities of the offspring. Cell division activities in the LRPs and LRs of the *AGL21*-overexpressing plants are much higher than that of the wild-type and mutant plants (Figure 10). Taken together, these results indicate that AGL21 positively regulates the auxin accumulation in the LRPs and LRs, thus stimulating the cell proliferation activity. So we could observe the phenotypes that *AGL21*-overexpressing lines had more and longer LRs than the wild-type, while the mutant had opposite phenotype in the root (Figures 1 and 6).

### *AGL21* Responds to Multiple External and Physiological Signals and Is Likely Involved in LR Development in Response to Environmental Constraints

It is generally believed that root is the main organ to collect signals and information from the environment and incorporate them into decisions about growth and development in order to adapt to the changing environmental conditions (Comstock, 2002; Lopez-Bucio et al., 2003; Malamy, 2005; Osmont et al., 2007). So far, several genes have been reported as possible regulators of RSA to various environment signals, such as N nutrient (Zhang and Forde, 1998; Malamy and Ryan, 2001; Engineer and Kranz, 2007; Krouk et al., 2010; Vidal et al., 2010), P nutrient (Ticconi et al., 2004; Svistoonoff et al., 2007), S nutrient (Kutz et al., 2002), and osmotic stress (Deak and Malamy, 2005; Yu et al., 2013). Besides external signals, plant development also responds to all kinds of internal signals, especially the plant hormones. In fact, plants can perceive and integrate many exogenous signals into the signaling pathways of plant hormones, resulting in root architecture change (Lopez-Bucio et al., 2002; Malamy, 2005; Achard et al., 2006; Jovanovic et al., 2007). However, the underlying mechanisms controlling root system development in response to different environmental constraints are not well understood.

In *Arabidopsis*, several root-expressed MADS-box TFs have been reported responding to N change in a manner similar to *ANR1*. *SOC1* was additionally found to respond to changes in the P and S supply (Gan et al., 2005). In addition, the *AGL17*-like clade genes in *Oryza sativa* had been found responding to osmotic stress, nitrate, and various hormonal treatments (Puig et al., 2013). Recently, the *XAL1/AGL12* and *XAL2/AGL14* genes had been proved to respond to auxin treatment (Tapia-Lopez et al., 2008; Garay-Arroyo et al., 2013). In our study, we found the expression of *AGL21* was induced by hormones, such as IAA, MeJA, and ABA (Figure 4). Moreover, we found many *cis*-acting elements in its promoter, including AuxRE, G-box, JARE, GCC-like box, and ABRE-like (Supplemental Figure 2), which are essential for genes responding to auxin, JA, and ABA, respectively (Menkens et al., 1995; Sessa et al., 1995; Grill and Himmelbach, 1998; Ulmasov et al., 1999; Xu and Timko, 2004). Through detailed analysis of *pAGL21::GUS* reporter line, we revealed that IAA and MeJA treatment could dramatically up-regulate the expression of *AGL21* in the root central cylinder as well as in the LRPs and LRs, even in the later-stage LRPs where *AGL21* is not expressed under normal conditions (Figure 4D and 4E). The elevated *AGL21* in these places may activate local auxin biosynthesis to favor LR development. However, ABA can increase *AGL21* expressed in the PR tip, proliferation zone, and elongation zone, but reduces its expression in the middle and top of the differentiation zone of the PR (Figure 4D and 4E). These results are consistent with the positive roles of auxin and JA and negative roles of ABA in LR development (Woodward and Bartel, 2005; Sun et al., 2009; Raya-Gonzalez et al., 2012). Therefore, our results indicate that AGL21 may be involved in LR development regulated by hormone signals which are stimulated by environmental cues.

In addition, *AGL21* expression also found responding to N and S starvations by qRT–PCR and GUS staining analyses (Figure 5). N deficiency is known to stimulate PR and particularly LR elongation but not LR initiation (Linkohr et al., 2002). We thus examine the root phenotypes of *AGL21*-overexpressing and mutant plants on N-rich and -free medium. The results showed that *AGL21*-overexpressing lines had longer LRs than wild-type plants under both these conditions, while LR growth was restrained in the mutant plants, especially under N-starved conditions. More specifically, under N-free conditions, LR length per cm PR had a slight increase in the wild-type and *AGL21*-overexpressing lines compared with that under N-rich conditions. However, compared with N-rich conditions, the LR length per cm PR of the mutant under N-free conditions apparently reduced (Figure 6E). Furthermore, the average LR length of the mutant decreased dramatically under N-free conditions compared with wild-type, while there was only a slight arrest under N-rich conditions, indicating that AGL21 plays some role in sustaining LR elongation in response to N availability. These results agree with the conference abstract of Suzuki et al. (2009), who reported that AGL21 is an essential factor to sustain LR growth under low-nitrate conditions. Therefore, our data demonstrate that AGL21 may play some role in N control of LR development. This is a good example of the function of AGL21 in LR development in response to environmental constraints. Furthermore, we also found *AGL21* expression was up-regulated by abiotic stresses, such as drought and high salinity (Figure 5C). Collectively, it is reasonable to propose that AGL21 may play some roles in the regulation of RSA plasticity in response to various environmental and intrinsic signals.

Taken together, our results show that *AGL21* expression can be induced by multiple environmental signals and internal hormones, and the up-regulated *AGL21* strengthens auxin accumulation in the LRPs and LRs by regulating local auxin biosynthesis in the root, thus increasing cell division activity and stimulating LR initiation and elongation. *AGL21* is also found positively regulated by auxin. Therefore, there is a positive feedback loop between auxin levels and auxin biosynthesis regulation via AGL21, which, in turn, affects auxin levels and distribution in the LPRs and LRs. As reported previously, auxin has been regarded as an integrator of diverse biotic and abiotic environmental signals and other hormonal signals to plant root development (Teale et al., 2008; Fukaki and Tasaka, 2009; Kazan, 2013; Lee and Cho, 2013). Hence, we propose that AGL21 may be a key factor to integrate the external and internal signals to auxin signals to regulate LRP initiation and LR growth, thus adapting to the environment more effectively. Our study also suggests that *AGL21* may be a promising candidate gene for improving RSA in crop improvement.

## METHODS

### Plant Material and Growth Conditions

Seeds were surface-sterilized for 10min in 15% bleach, washed five times with sterile water, stratified at 4°C for 2 d, and plated on MS solid medium containing 1% (w/v) sucrose and 0.6% (w/v) agar at 22°C under 16-h light/8-h dark photoperiod. N-free medium was based on MS basal salt solution by replacing 20mM KNO_3_ and 20mM NH_4_NO_3_ with 20mM KCl. S-free medium was prepared as described previously (Wu et al., 2010).

### Identification of the *AGL21* Knockout Mutants

Two T-DNA insertion lines (CS118325 and GK_157C08) were obtained from the Arabidopsis Biological Resource Center (ABRC). CS118325 homozygotes were identified by genomic PCR with three primers: Spm32, CS118325 LP, and CS118325 RP. The homozygous mutant plants were confirmed by RT–PCR using gene-specific primers *AGL21* LP and *AGL21* RP, and *β-Tubulin8* (*TUB*) was used as control with specific primers. All the primers used are shown in Supplemental Table 1.

### Constructs and Generation of Transgenic Plants

For generation of *AGL21*-overexpressing plants, the *35S::AGL21* overexpression construct was made by inserting the coding region of *AGL21* amplified by PCR using *AGL21-*attb-LP and *AGL21*-attb-RP into pCB2004 (Lei et al., 2007) via the GATEWAY cloning system. For promoter analysis, a *pAGL21::GUS* construct was produced by inserting a 2.6-kb promoter fragment amplified using forward primer *AGL21*-Pro-LP and reverse primer *AGL21*-Pro-RP into pCB308R (Lei et al., 2007). For protein localization, an *AGL21* full-length coding sequence amplified by RT–PCR using specific primers *AGL21*-attb-LP1 and *AGL21*-attb-RP1 was inserted between the 35S promoter and EGFP sequences in pGWB5 (Nakagawa et al., 2007) to get *pGWB5::AGL21* by the GATEWAY cloning system. To get the native promoter–gene fusion construct, a fragment containing *AGL21* promoter and coding region amplified by genomic PCR with primers *AGL21*-attb-LP2 and *AGL21*-attb-RP2 was cloned into pMDC110 to fuse with GFP (Curtis and Grossniklaus, 2003). All the primers used are shown in Supplemental Table 1.

The constructs described were used to transform *Arabidopsis* using the *Agrobacterium*-mediated floral-dip method (Clough and Bent, 1998). Glufosinate-resistant T_2_ transgenic plants were obtained for functional analysis.

### Quantitative Real-Time PCR (qRT–PCR)

qRT–PCR was performed as described previously (Yu et al., 2013). The transcript levels of *AGL21* were examined using specific primers *AGL21-*qPCR LP and *AGL21-*qPCR RP. *UBQ5* was used as the internal control, using specific primers *UBQ5* LP and *UBQ5* RP. All the primers used are shown in Supplemental Table 1. The results were based on the average of three parallel experiments.

### Histochemical Detection of GUS Activity and GFP Imaging

The GUS activity staining was conducted as described previously (Xi et al., 2012). After incubating at 37°C for 2–12h in the dark, individual representative seedlings were photographed. Fluorescence of GFP in the transgenic plants was observed using a confocal microscope (Carl Zeiss LSM710, www.leica.com/).

### Morphological Characterization of Roots

Root morphology was examined on MS medium solidified with 0.6% agar. Briefly, seeds were germinated on MS medium and 5-day-old seedlings were transferred to MS medium, MS medium containing hormones, or other nutrition lacking medium plates and grown vertically for a few days. Visible LR number was counted every day from the transfer day, and pictures of the plate were taken. Digital images of plants were used for root length measurement by hand using ImageJ software (NIH). LRPs and LRs of *DR5:GUS* and *pCYCB1;1::GUS* plants in different genetic backgrounds were photographed or counted using HIROX’s KH-7700 digital microscope. Classification of LRP developmental stages was performed according to Malamy and Benfey (1997).

### Hormone and Abiotic Stress Treatments

For the qRT–PCR experiment, 8-day-old plants grown on MS agar medium were transferred to MS nutrient solution supplemented with hormones or N/S-free nutrient solution for different times as indicated and harvested for RNA extraction. Drought and high-salinity treatments were carried out as reported previously (Seki et al., 2002).

For GUS staining analysis, 7-day-old plants grown on MS agar medium were transferred to MS agar medium supplemented with hormones or N/S-free agar medium for the indicated time points and harvested for GUS staining.

### IAA Content Measurement

The free total IAA content was measured by ELISA as described by Lin et al. (2005).

## SUPPLEMENTARY DATA

Supplementary Data are available at *Molecular Plant Online.*

## FUNDING

This work was supported by the Chinese Academy of Science (grant no. KSCX3-YW-N-007), the Ministry of Science and Technology of China (grant no. 2012CB114304), and the National Nature Science Foundation of China (grant no. 30830075).

## Supplementary Material

Supplementary Data
