# Supplementary material for: MADS-Box Transcription Factor AGL21 Regulates Lateral Root Development and Responds to Multiple External and Physiological Signals
Source: Mol Plant. 2014 Aug 13;7(11):1653–69. doi: 10.1093/mp/ssu088 (PMC4228986; doi:10.1093/mp/ssu088)
Supplement: Supplementary Data [file supp_7_11_1653__index.html]

MADS box transcription factor AGL21 regulates lateral root development and responds to multiple external and physiological signals — MADS-Box Transcription Factor AGL21 Regulates Lateral Root Development and Responds to Multiple External and Physiological Signals — MADS-Box Transcription Factor AGL21 Regulates Lateral Root Development and Responds to Multiple External and Physiological Signals — MADS-Box Transcription Factor AGL21 Regulates Lateral Root Development and Responds to Multiple External and Physiological Signals — Supplementary Data 

# MADS-Box Transcription Factor AGL21 Regulates Lateral Root Development and Responds to Multiple External and Physiological Signals

## Supplementary Data

Data files

**Files in this Data Supplement:**

- Supplementary Data - Supplementary Data
