# Supplementary material for: MADS-Box Transcription Factor AGL21 Regulates Lateral Root Development and Responds to Multiple External and Physiological Signals
Source: Mol Plant. 2014 Aug 13;7(11):1653–69. doi: 10.1093/mp/ssu088 (PMC4228986; doi:10.1093/mp/ssu088)
Supplement: Supplementary Data [file supp_ssu088_Supporting_Information_for_Yu___et___al__final_.pdf]

Supporting Information for MADS box transcription factor AGL21 regulates lateral root development and responds to multiple external and physiological signals by Yu et al.

Supplemental Figure S**1.** Expression pattern of *DR5:GUS* at LRP initiation sites of representative wild type, *35S::AGL21*and *agl21* mutant roots grown on MS medium. Photographs show typical roots of 8-day-old seedlings. Arrows show LRP or emerged LR, as indicated by *DR5:GUS* staining.

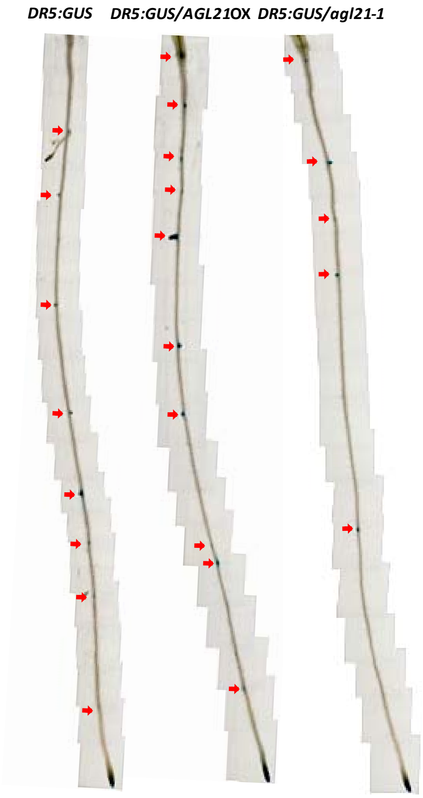

Supplemental Figure S**2. Distribution of *cis*-acting elements in the promoter region of *AGL21*.**

Auxin response elements (AuxRE, TGTCxC) and ABA response elements (ABRE, ACGTGG/TC) are important for auxin and ABA response genes to response to these hormones (Grill and Himmelbach, 1998; Ulmasov et al., 1999). To explore the possibility that auxin and ABA signaling directly induced transcription of *AGL21*, we screened the promoter of *AGl21* for the AuxRE and ABRE motifs. We found 3 AuxRE

and 1 ABRE-like *cis* elements in the *AGL21* promoter. We also found 4 G-box, which play a role in the responsiveness of plant promoters to anaerobiosis, light and plant hormones such as ethylene, ABA and JA (Menkens et al., 1995). Moreover, the promoter of *AGL21* also contains two other *cis*-acting elements, GCC-box like and JARE, which was reported participating in ethylene- and JA-induced gene expression, respectively (Sessa et al., 1995; Seo et al., 2013).

AuxRE: TGTCxC, ABRE-like: (C/G/T)ACGTG(G/T)(A/C), GCC-box like: TGCGCCC, JARE: G/CTCCTGA, G-box: CACGTG

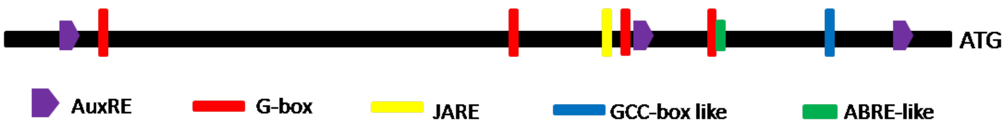

Supplemental Figure S3. IAA strongly induced *AGL21* expression in the LRPs and LRs.

7-day-old seedlings of *pAGL21::GUS* transgenic line grown on MS agar medium were transferred either to hormone-free MS agar medium or to MS agar medium supplemented with 10 μM IAA for 1d and then harvested for GUS staining for 8 hours.

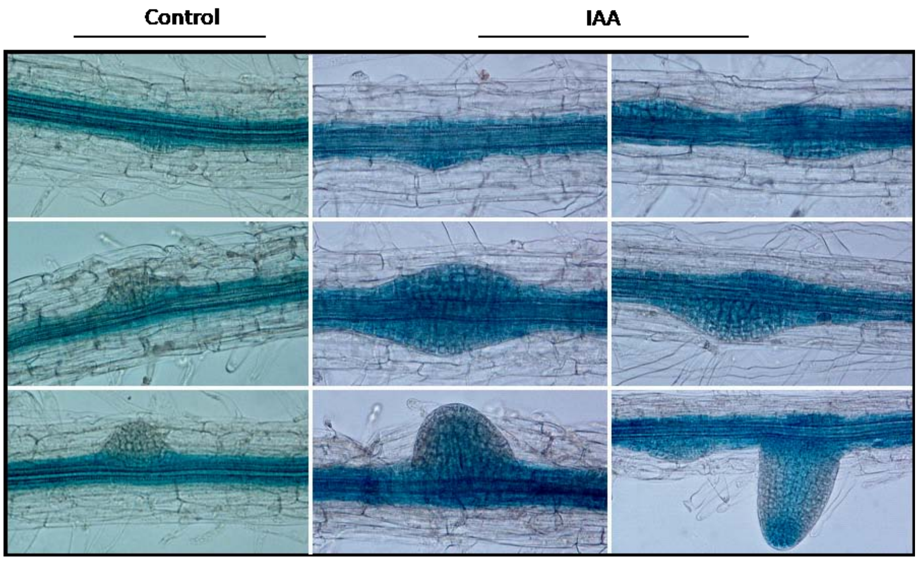

Supplemental Figure S4. AGL21 increased the *DR5:GUS* expression in leaves.

9-day old plants grown on MS medium were used for GUS staining for 6 hours.

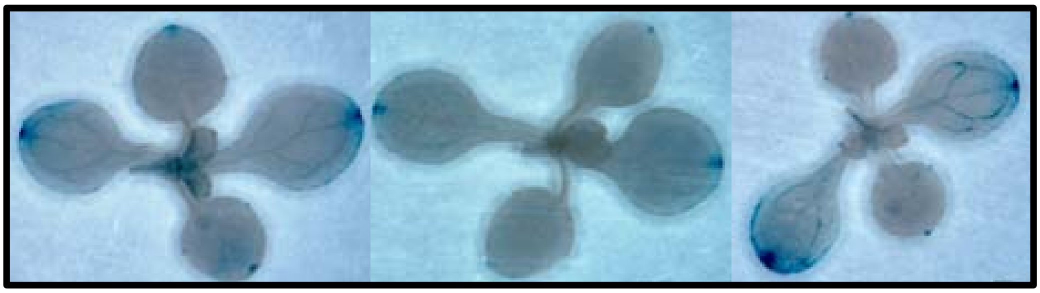

Supplemental Table 1. Primers used for PCR

Name	Sequence (5’ to 3’)
Spm32	5’-TACGAATAAGAGCGTCCATTTTAGAGTGA-3’
CS118325 LP	5’-GAAGAACCATGCTGCTTATCG-3’
CS118325 RP	5’-ACTATTGAACCCCGCATCAG-3’
AGL21 LP	5’-ATGAGGAAATCCAAAGCTAAT-3’
AGL21 RP	5’-TACTTTCTGATCAACTGATAAAGG-3’
TUB LP	5’-CTTAAGCTCACCACTCCAAGCT-3’
TUB RP	5’-GCACTTCCACTTCGTCTTCTTC-3’
AGL21-attb-LP	5’-GGGGACAAGTTTGTACAAAAAAGCAGGCT ATGAGGAAATCCAAAGCTAAT-3’
AGL21-attb-RP	5’-GGGGACCACTTTGTACAAGAAAGCTGGGT TACTTTCTGATCAACTGATAAAGG-3’
AGL21-Pro-LP	5’-GGGGACAAGTTTGTACAAAAAAGCAGGCT CATTCACACTACCACTCTGATTACC-3’
AGL21-Pro-RP	5’-GGGGACCACTTTGTACAAGAAAGCTGGGT CAATTTTATCCTCTAATTGAATCTCCT-3’
AGL21-attb-LP1	5’-GGGGACAAGTTTGTACAAAAAAGCAGGCT ATGAGGAAATCCAAAGCTAAT-3’

AGL21-attb-RP1	5’-GGGGACCACTTTGTACAAGAAAGCTGGGT<br>CTTCGTTTGCTCTTGGTGGAGTGTC-3’
AGL21-attb-LP2	5’-GGGGACAAGTTTGTACAAAAAAGCAGGCT<br>TTGGTAAAGAAATAGGTCATCTGTG-3’
AGL21-attb-RP2	5’-GGGGACCACTTTGTACAAGAAAGCTGGGT<br>TTCGTTTGCTCTTGGTGGAGTGTC-3’
AGL21-qPCR LP	5’-CTTCATGCTGGAGCTTGCAAAGTC-3’
AGL21-qPCR RP	5’-AGCTATTCTCTGTGATGCCGAGGT-3’
UBQ5 LP	5’-AGAAGATCAAGCACAAGCAT-3’
UBQ5 RP	5’-CAGATCAAGCTTCAACTCCT-3’
PIN1 qPCR LP	5’-GGTCGGAACTCTAACTTTGGT-3’
PIN1 qPCR RP	5’-CAGCTCCAGCAGCAGTTCCA-3’
PIN2 qPCR LP	5’-TATATTCGGAATGCTGGTTGCTTTG-3’
PIN2 qPCR RP	5’-CCATACACCTAAGCCTGACCTGGAA-3’
PIN3 qPCR LP	5’-GAGGGAGAAGGAAGAAAGGGAAAC-3’
PIN3 qPCR RP	5’-CTTGGCTTGTAATGTTGGCATCAG-3’
PIN4 qPCR LP	5’-GTTGTCTCTGATCAACCTCGAAA-3’
PIN4 qPCR RP	5’-TATCAAGACCGCCGATATCATC-3’
PIN7 qPCR LP	5’-CCAAGATTAGTGGAACGCAAC-3’
PIN7 qPCR RP	5’-GAAAAGGGTTTTTGGATCCTC-3’
AUX1 qPCR LP	5’-AGTAGCAAATGACAACGGAACAG-3’
AUX1 qPCR RP	5’-AGAGCCACCGTGCCATAGG-3’
ASA1 qPCR LP	5’-ATGTCTTCCTCTATGAACGTAGC-3’
ASA1 qPCR RP	5’-ACAGCGGTAAATTGGTATAAGG-3’
PAT1 qPCR LP	5’-ATGGTTATTGCGGTGGCGAC-3’
PAT1 qPCR RP	5’-ATCGTCGCCGACTCAATGTC-3’
AMI1 qPCR LP	5’-GCTGCTGCAGGAGAACGCAACC-3’
AMI1 qPCR RP	5’-GCTGCTGCAGGAGAACGCAACC-3’
AAO1 qPCR LP	5’-TGCCTGTTCCAGCAACAATG-3’

AAO1 qPCR RP	5’-TAAGCAGAACACCGCCATTG-3’
TAA1 qPCR LP	5’-GACTCCTTAGACACACCAATCGAGTTC-3’
TAA1 qPCR RP	5’-GACTCCTTAGACACACCAATCGAGTTC-3’
TAR2 qPCR LP	5’-CATGATTTGGCTTACTATTGGCCACAG-3’
TAR2 qPCR RP	5’-GTCTTTCACCAAAGCCCATCCAATC-3’
TAR3 qPCR LP	5’-GCTGGGCATTAGTAAAAGAGAAAACTG-3’
TAR3 qPCR RP	5’-CGAAAAACGCGTAGACATGGAAAA-3’
YUC1 qPCR LP	5’-TGGAGAGTAAAGACTCATGAT-3’
*YUC1 q*PCR RP	5’-GTACTCACTCGCGTGAACGAT-3’
YUC2 qPCR LP	5’-GGTGACACGGATCGGTTAGGGT-3’
YUC2 qPCR RP	5’-TGCCGAATAATGCATTACCCGT-3’
YUC3 qPCR LP	5’-CTTGAGATTGATTCCGTTATTC-3’
YUC3 qPCR RP	5’-GGAGAAGAAGTCGTTGTC-3’
YUC5 qPCR LP	5’-ATCTCGCAAATCACAATG-3’
YUC5 qPCR RP	5’-ACCACTTCATCAACATCA-3’
YUC6 qPCR LP	5’-TATACGCGGTCGGATTCACA-3’
YUC6 qPCR RP	5’-CCACCACAATCACTCTCACT-3’
YUC7 qPCR LP	5’-ATGTATTACCGAGAGAAGTTC-3’
YUC7 qPCR RP	5’-TTGTGAGAACGAGAAGAGTC-3’
YUC8 qPCR LP	5’-TGGTTG GAAAGGAAGGACAGG-3’
YUC8 qPCR RP	5’-TCGTGGGTTGTTTTGTTTCAAG-3’
YUC9 qPCR LP	5’-ATCTTGCTAACCACAATG-3’
YUC9 qPCR RP	5’-CCACTTCATCATCATCAC-3’
NIT1 qPCR LP	5’-GGCGTTCATAACGAAGAAGGGCGTG-3’
NIT1 qPCR RP	5’-TTCCTTCTCTATGGCTCCCATTACC-3’
NIT3 qPCR LP	5’-AGCGAAGTTGGTGTTGTTTCCC-3’
NIT3 qPCR RP	5’-CCAACTCAGCCAATCTTTCCAC-3’
NIT4 qPCR LP	5’-GCCTTCTTTGAGAACCGCAATGTATGCC-3’
NIT4 qPCR RP	5’-CAAGTG CAATATGAGTCATTGATGCTAGCCAAG-3’

References

Grill E, Himmelbach A (1998) ABA signal transduction. Curr Opin Plant Biol **1:** 412-418

Menkens AE, Schindler U, Cashmore AR (1995) The G-box: a ubiquitous regulatory DNA element in plants bound by the GBF family of bZIP proteins. Trends Biochem Sci **20:** 506-510

Seo JS, Koo YJ, Jung C, Yeu SY, Song JT, Kim JK, Choi Y, Lee JS, Do Choi Y (2013) Identification of a novel jasmonate-responsive element in the AtJMT promoter and its binding protein for AtJMT repression. Plos One **8:** e55482

Sessa G, Meller Y, Fluhr R (1995) A GCC element and a G-box motif participate in ethylene-induced expression of the PRB-1b gene. Plant Mol Biol **28:** 145-153

Ulmasov T, Hagen G, Guilfoyle TJ (1999) Dimerization and DNA binding of auxin response factors. Plant J **19:** 309-319
